# Effects of Dietary Supplementation of Carnosine on Mitochondrial Dysfunction, Amyloid Pathology, and Cognitive Deficits in 3xTg-AD Mice

**DOI:** 10.1371/journal.pone.0017971

**Published:** 2011-03-15

**Authors:** Carlo Corona, Valerio Frazzini, Elena Silvestri, Rossano Lattanzio, Rossana La Sorda, Mauro Piantelli, Lorella M. T. Canzoniero, Domenico Ciavardelli, Enrico Rizzarelli, Stefano L. Sensi

**Affiliations:** 1 Molecular Neurology Unit, Center of Excellence on Aging (Ce.S.I.), University “G. d'Annunzio”, Chieti-Pescara, Italy; 2 Department of Neuroscience and Imaging, University “G. d'Annunzio”, Chieti-Pescara, Italy; 3 Department of Biological and Environmental Science, University of Sannio, Benevento, Italy; 4 Department of Oncology and Neuroscience, University “G. d'Annunzio”, Chieti-Pescara, Italy; 5 Department of Chemistry, University of Catania, Catania, Italy; 6 Department of Neurology, University of California Irvine, Irvine, California, United States of America; Brigham and Women's Hospital, Harvard Medical School, United States of America

## Abstract

**Background:**

The pathogenic road map leading to Alzheimer's disease (AD) is still not completely understood; however, a large body of studies in the last few years supports the idea that beside the classic hallmarks of the disease, namely the accumulation of amyloid-β (Aβ) and neurofibrillary tangles, other factors significantly contribute to the initiation and the progression of the disease. Among them, mitochondria failure, an unbalanced neuronal redox state, and the dyshomeostasis of endogenous metals like copper, iron, and zinc have all been reported to play an important role in exacerbating AD pathology. Given these factors, the endogenous peptide carnosine may be potentially beneficial in the treatment of AD because of its free-radical scavenger and metal chelating properties.

**Methodology:**

In this study, we explored the effect of L-carnosine supplementation in the 3xTg-AD mouse, an animal model of AD that shows both Aβ- and tau-dependent pathology.

**Principal Findings:**

We found that carnosine supplementation in 3xTg-AD mice promotes a strong reduction in the hippocampal intraneuronal accumulation of Aβ and completely rescues AD and aging-related mitochondrial dysfunctions. No effects were found on tau pathology and we only observed a trend toward the amelioration of cognitive deficits.

**Conclusions and Significance:**

Our data indicate that carnosine can be part of a combined therapeutic approach for the treatment of AD.

## Introduction

Mitochondria-driven overproduction of reactive oxygen species (ROS) and imbalanced homeostasis for endogenous metals and zinc (Zn^2+^) in particular, are important co-factors in the development and progression of several neurological disorders, including Alzheimer's Disease (AD; [1], [2]). Furthermore, a growing body of evidence indicates that mitochondrial failure is an early event in AD, suggesting that, along with deposits of amyloid-β (Aβ) and hyperphosphorylated tau (h-tau) protein, the malfunctioning of the organelles plays a synergistic role in triggering the neuronal death and cognitive decline associated with the disease (Reviewed in [3]). For instance, enhanced ROS generation of mitochondrial origin [4], [5] can greatly interfere with homeostatic mechanisms regulating levels of intracellular free Zn^2+^ ([Zn^2+^]_i_), thereby producing intraneuronal [Zn^2+^]_i_ rises that generate a vicious loop leading to enhanced Zn^2+^-dependent formation of ROS as well as Aβ oligomerization [2], [6], [7], [8].

The rationale for addressing Zn^2+^ dyshomeostasis in AD is substantial [9]. In vitro, Zn^2+^ induces the aggregation and oligomerization of Aβ [6] and, in AD transgenic animals, *in vivo* release of pre-synaptic Zn^2+^ from glutamatergic terminals promotes amyloid plaque formation [10]. Moreover, recent findings in cultured neurons from triple-transgenic AD mice, the 3xTg-AD, overexpressing mutant amyloid precursor protein (APP), presenilin 1 (PS1), and h-tau indicate that the pro-AD environment fostered by these mutants enhances ROS generation and ROS-mediated [Zn^2+^]_i_ mobilization from intracellular Zn^2+^- binding proteins, thereby providing a potential mechanism for the initiation of the intraneuronal aggregation of Aβ [11], [12].

Finally, treatment with Zn^2+^ and Cu^2+^ chelators like clioquinol (CQ) and its derivative, PBT2, shows efficacy in reducing amyloid plaques and counteracts cognitive deficits in AD transgenic mice [13], [14]. Furthermore, recent clinical trials employing CQ or PBT2 have found that the compounds are also effective in protecting against the development and progression of cognitive deficits in AD patients [15], [16].

Carnosine (β-alanyl-L-histidine) is a dipeptide found at high concentrations in glial and neuronal cells throughout the brain [17]. The functional role of carnosine is still not completely understood; however, several studies indicate that the dipeptide exerts protective actions against metal- and or Aβ-mediated toxicity by acting as anti-oxidant and free-radical scavenger [18], [19], [20], [21]. Moreover, because of its enrichment in histidine residues, carnosine has also been proposed as a chelator for divalent cations like Cu^2+^ and Zn^2+^. Furthermore, most likely because of its chelating properties, carnosine can relieve the Zn^2+^-mediated inhibition of NMDA and GABA receptors and therefore modulate neuronal excitability [22]. Carnosine also exerts anti-aging activities by neutralizing injurious glycated proteins and aldehydic products of lipids peroxydation (i.e., acetaldehyde, malondialdehyde, and hydroxynonenal), thereby attenuating the toxicity of these bioproducts and preventing the cross-linking of glycoxidised proteins to physiological macromolecules [23], [24], [25], [26]. In the context of AD, carnosine has been suggested as therapeutic agent given its capability to act as a metal chelator, free radical scavenger as well as an inhibitor of Aβ toxicity [20]. However, one possible limitation in employing carnosine as an anti-AD drug is that brain carnosine is rapidly inactivated by the activity of three different isoforms of the carnosine degrading enzyme, carnosinase. Increased carnosinase activity has in fact been found in AD patients as well as in aging individuals [27], [28] and decreased plasmatic levels of carnosine have been reported in AD patients [29].

In this study we investigated the potential beneficial effects of dietary carnosine supplementation (10 mM in drinking water) in 3xTg-AD mice, an AD animal model that develops amyloid- and tau-dependent pathology as well as AD-related cognitive deficits [11], [30].

## Results

### Carnosine chelates intracellular Zn^2+^

**Figure 1:**
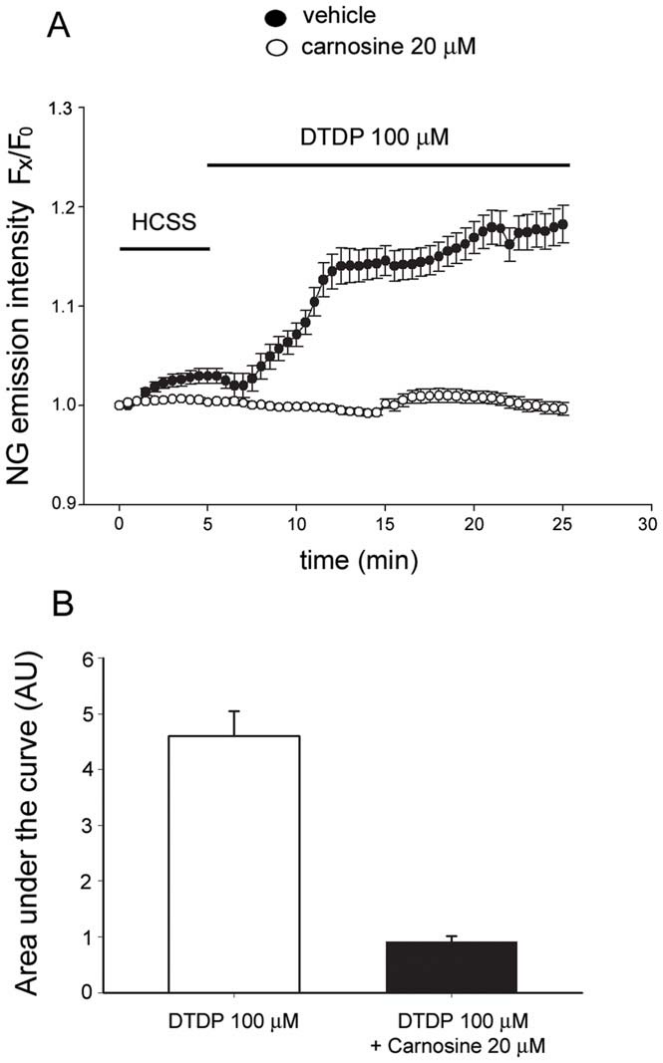
Carnosine chelates [Zn^2+^]_i_ rises mobilized from intracellular sites. (A) Time course of DTDP-mediated [Zn^2+^]_i_ rises in cortical glial cells. Newport Green loaded astrocytes (white circle) were imaged upon a 20 min incubation in a physiological buffer, HCSS, and during a 20 min exposure to DTDP (100 µM), a compound that mobilizes Zn^2+^ from intracellular Zn^2+^-binding proteins. In parallel experiments, Newport Green loaded glial cultures (black circles) were incubated in HCSS plus carnosine (20 mM) for 20 min and subsequently exposed to DTDP plus carnosine for other 20 min. The graph shows the time course of DTDP-induced Newport Green fluorescence changes (expressed as ratio of F_X_/F_0_) in carnosine treated and untreated glial cultures. Traces show mean (± SEM) fluorescence changes deriving from 3 different experiments for each condition. (B) Bar graph depicts the overall cytosolic [Zn^2+^]_i_ rise expressed as area under the curve after the DTDP exposure. (*) indicates differences between control and carnosine treated astrocytes (*p*<0.001).

Carnosine has been described to form complexes with Zn^2+^ in aqueous solution [31], [32]; however, to date, no “in situ” experiments have demonstrated whether the dipeptide can chelate Zn^2+^ in cellular models. Thus, to evaluate the, *in vitro,* chelating properties of the peptide, we loaded cultured cortical glial cells with the Zn^2+^-sensitive fluorescent probe, Newport green (K_d_ for Zn^2+^ = 1·10^−6^ M), and studied whether the addition of carnosine would decrease [Zn^2+^]_i_ rises triggered by oxidative stress. We chose to use a relatively high carnosine concentration (20 mM) because of the nature of the employed paradigm where we triggered acute and intense [Zn^2+^]_i_ rises. In the first set of experiments, Newport Green loaded cells were, after baseline evaluation, exposed for 20 min to the disulfide oxidizing agent 2,2′-dithiodipyridine [(DTDP; 100 µM), a compound that promotes [Zn^2+^]_i_ release from Zn^2+^-binding proteins like metallothioneins (MTs; [33], [34]). [Zn^2+^]_i_ rises were evaluated over a period of 20 min (Figure 1). As expected, DTDP exposure caused a sustained increase of [Zn^2+^]_i_ levels. However, when the same experiment was repeated in cultures treated with 20 mM carnosine during the basal period as well as during the DTDP exposure, ROS-driven cytosolic [Zn^2+^]_i_ rises were largely attenuated, confirming the idea that carnosine is an effective cell permeable [Zn^2+^]_i_ chelator (Figure 1).

### Carnosine shows sub-maximal effects in rescuing long-term memory deficits in treated 3xTg-AD mice

**Figure 2:**
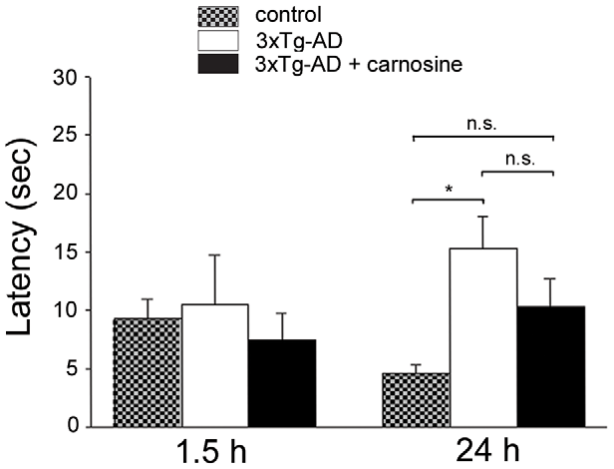
Carnosine treatment has sub-maximal effect in counteracting memory deficits in 3xTg-AD mice. Carnosine-treated (n = 9) and untreated 3xTg-AD (n = 13) as well as control mice (n = 11) were tested for the spatial memory version of the MWM. Mice were given a memory probe trial where the platform was removed 1.5 and 24 h after the last training trial to evaluate short-term and long-term memory, respectively. All the three groups showed no short-term memory impairment as assessed by MWM performance at 1.5 h. When tested for long-term memory deficits at 24 h, untreated 3xTg-AD mice exhibited a significant deficit in their performance as assessed by the marked increase in the time (latency) they employed to reach the point where the platform used to be. Carnosine-fed mice showed a trend toward decreased long-term memory deficit. Error bars are shown mean (± SEM); (*) indicates p<0.05.

3xTg-AD mice have been reported to show age-dependent spatial memory decline as early as 5–6 months of age (m.o.a.; [11], [30]). We designed our study to test the effect of carnosine supplementation on AD-like memory deficits of 3xTg-AD mice. We chose to use 10 mM carnosine, moving from the assumption that the AD-related Zn^2+^ dyshomeostasis is a chronic process that is likely to be associated with less intense rises in cation levels compared to what we have described in the Zn^2+^ imaging experiments). To evaluate the cognitive performance of treated and control mice, we employed the Morris Water Maze (MWM) test. Animals were subjected to 3 consecutive training days for the hidden-platform version of the MWM, a task that measures hippocampus-dependent spatial memory [35]. At first, we assessed the integrity of learning processes and found no differences in task acquisition (data not shown), indicating that all groups learned the same way to find a submerged platform using intra- and extra-maze visible cues. After the last training trial, spatial reference memory probe trials were conducted at 1.5 and 24 h in order to evaluate short- and long-term memory performances, respectively. As expected, compared to control (PS1KI) animals [30], 3xTg-AD mice showed no impairment in short-term memory while they manifested long-term memory deficits as indicated by the statistically significant increase in the time spent to find the platform (Figure 2). Carnosine supplementation was not able to completely rescue long-term memory deficits in treated 3xTg-AD mice. We observed a positive trend toward a better cognitive performance as indicated by the decreased latency to find the platform. Untreated 3xTg-AD mice showed a statically significant worst performance (p<0.05), no differences were found when the comparison was made between treated 3xTg-AD and control mice (p<0.1); however, when treated and untreated 3xTg-AD mice were compared, the difference failed to reach statistical significance (Figure 2).

### Carnosine supplementation reduces intraneuronal Aβ deposition but is ineffective on tau pathology in the hippocampus of 3xTg-AD mice

**Figure 3:**
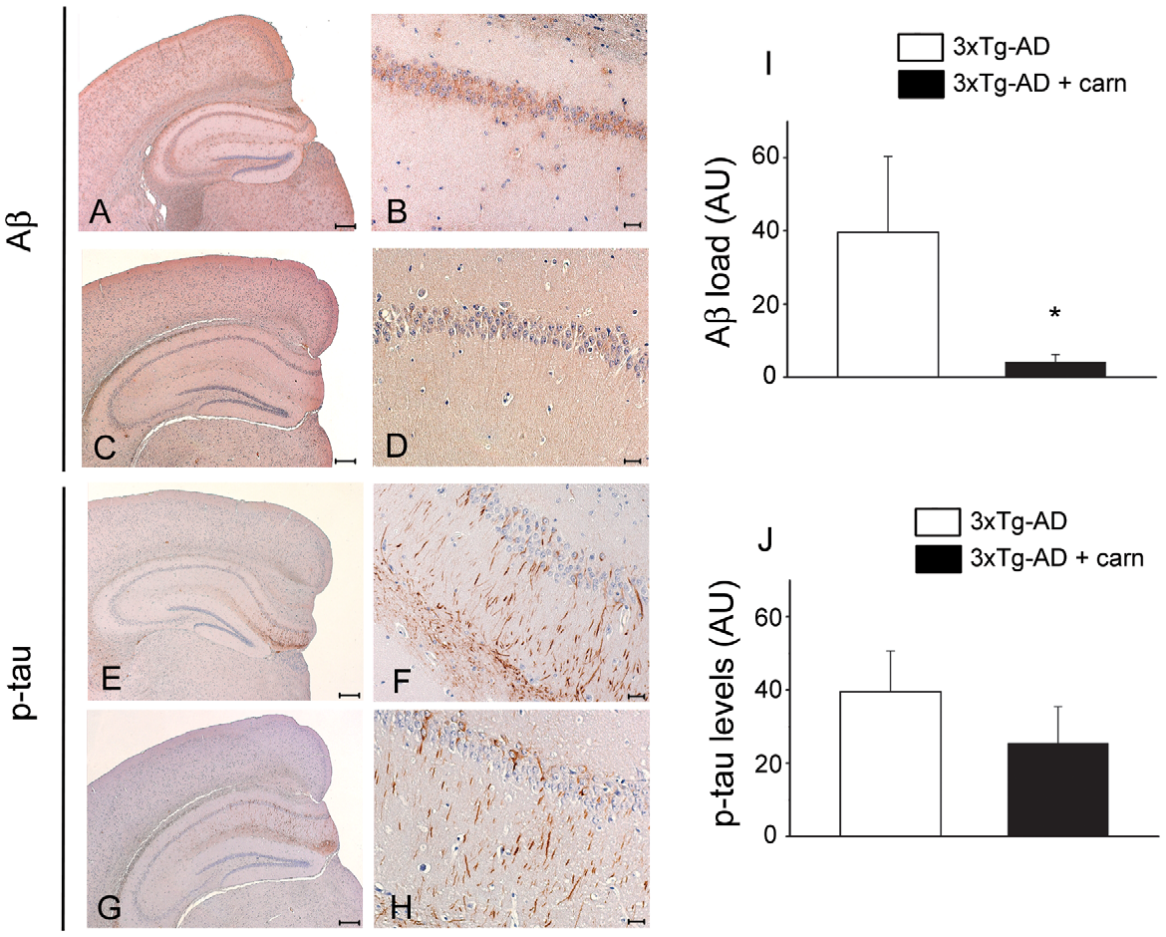
Carnosine supplementation reduces intraneuronal Aβ but not tau accumulation in the hippocampus of 3xTg-AD mice. Immunohistochemistry was employed to detect deposits of intraneuronal Aβ (A–D) and h-tau (E–H) in brain slices from treated (n = 3) and untreated (n = 3) 3xTg-AD mice (left column: 5× magnification, scale bar 200 µm; right column: 40× magnification of the hippocampal CA1 subregion, scale bar 20 µm). Compared to untreated 3xTg-AD mice (A,B), immunohistochemical staining shows a strong decrease of intraneuronal Aβ immunoreactivity in the hippocampus of carnosine treated 3xTg-AD mice (C,D). (I) Quantification of intraneuronal Aβ load as shown in B and D. Untreated 3xTg-AD mice (E,F) show comparable intraneuronal h-tau deposits in the hippocampus compared to treated mice (G,H). (J) Quantification of h-tau levels as shown in F and H. Error bars are shown as mean (± SEM); (*) indicates p<0.05.

After cognitive evaluation, mice were killed and neuropathology assessed. 3xTg-AD mice have been reported to undergo a progressive intraneuronal accumulation of Aβin AD-relevant regions starting at 4 m.o.a. [11]. To investigate whether carnosine supplementation can decrease the brain Aβ load, immunohistochemistry was performed with the anti-Aβ DE2B4 primary antibody and analysis of this assay showed a significant decrease of intraneuronal Aβ in the hippocampus of treated 3xTg-AD mice (Figure 3A-D).

At about 12 m.o.a., 3xTg-AD mice also develop extensive neurofibrillary tangles, first in the hippocampus (in particular within pyramidal neurons of the CA1 subfield) and then in the cortex [11]. To investigate the effect of carnosine on the development of tau pathology we employed an anti-tau AT180 primary antibody that specifically detects tau phosphorylation at the thr231/ser235 site. Diffuse phospho-tau immunoreactivity was found in the CA1 subfield of untreated 3xTg-AD mice (Figure 3E,G). Interestingly, although carnosine was able to reduce the amyloid load, the treatment produced no decreases of phospho-tau immunoreactivity in the 3xTg-AD CA1 subfield (Figure 3E-H), indicating that the peptide has no effect on tau pathology.

### Carnosine supplementation counteracts age-dependent mitochondrial deficits

**Figure 4:**
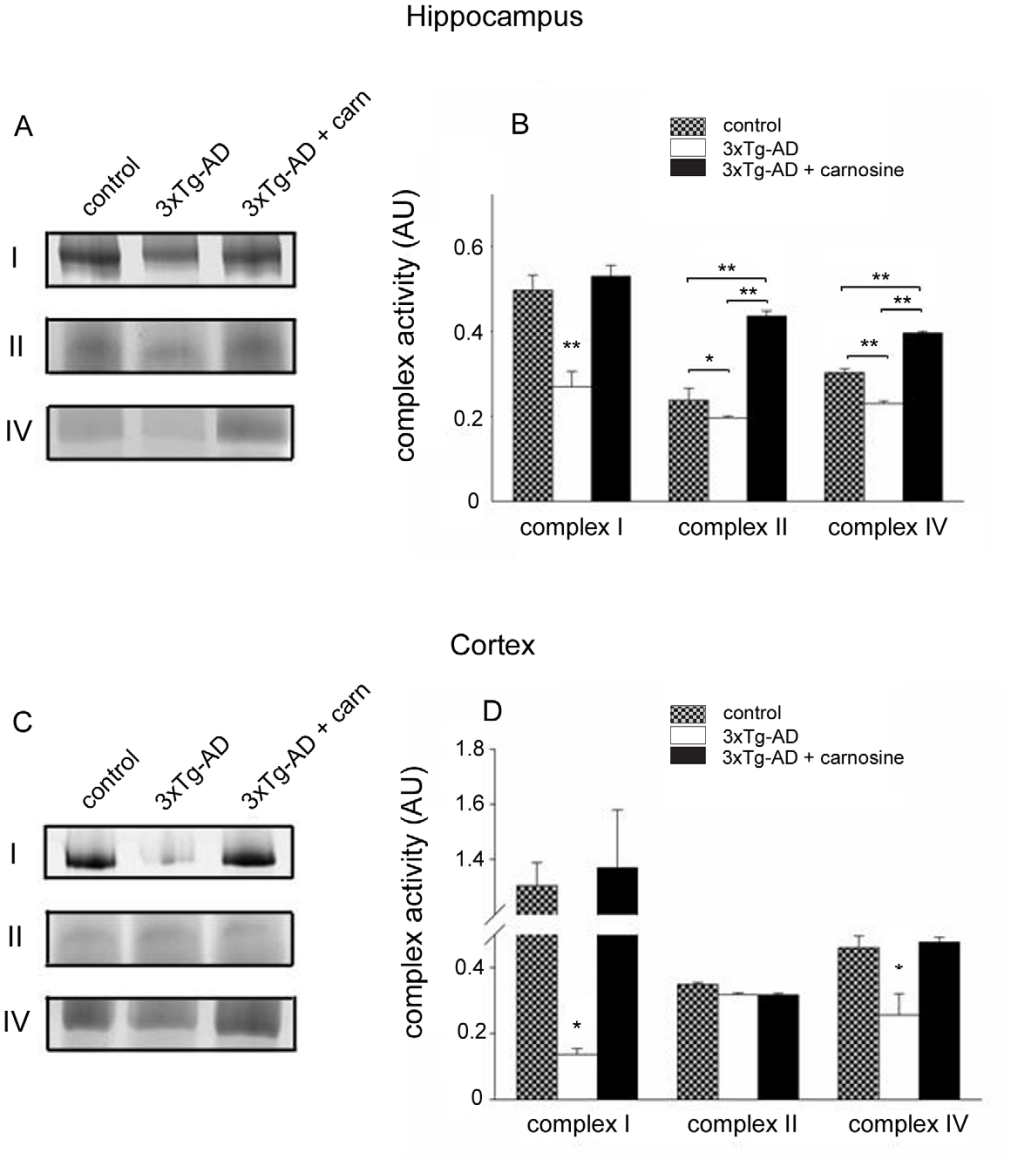
Carnosine supplementation rescues mitochondria deficits in 3xTg-AD mice. BN-PAGE was employed to assess the activity of mitochondrial complexes I, II, and IV in isolated mitochondria obtained from the hippocampus and cerebral cortex of control (PS1KI), untreated, and carnosine-treated 3xTg-AD (n = 3 to 5) mice at 12–14 m.o.a. (A,B) When compared with age-matched untreated mice, activity of mitochondrial complexes I and IV are found decreased in the hippocampus of 3xTg-AD mice while carnosine supplementation completely prevents the deficits. (C,D) When comparing complex activities of mitochondria obtained from the cortex of 3xTg-AD vs age-matched control mice, complex I and IV are found strongly compromised and carnosine treatment rescued these deficits. Error bars are expressed as mean (± SEM); (*) indicates p<0.05; (**) indicates p<0.01.

Previous studies have shown that brain mitochondria of 3xTg-AD mice show deficits in mitochondrial respiration [5]. We have also found a similar age-dependent decrease in the activity of complex I (NADH-dehydrogenase), II (succinate-dehydrogenase), and IV (COX) in mitochondria isolated from the cortex and hippocampus of 3xTg-AD animals [36]. To test the effect of carnosine on such age-dependent mitochondrial deficits, treated and untreated 3xTg-AD mice were investigated for the activity of mitochondrial complexes I, II, and IV by employing a combination of blue-native poliacrylammide gel electrophoresis (BN-PAGE) and subsequent histochemical in-gel staining of isolated mitochondria from the hippocampus and cerebral cortex. Data from these experiments indicate that, compared to control mice, hippocampal mitochondria of untreated 3xTg-AD mice show a strong deregulation in the activity of complexes I, II, and IV (Fig. 4A–B). Interestingly, carnosine-fed 3xTg-AD mice exhibited a complete recovery of all these deficits and in the case of complexes II and IV the activity was actually significantly higher compared to mitochondria of control animals (Figure 4A–B). Analysis of mitochondrial activity in the cortex revealed that untreated 3xTg-AD mice showed a dramatic decline in the activity of complex I and, to a lesser extent, of complex IV (Figure 4C–D). Similarly to what was found in the hippocampus, carnosine treatment promoted a complete recovery of all these cortical deficits.

## Discussion

Carnosine is an endogenous dipeptide highly expressed throughout the brain that has been suggested as a therapeutic tool in the treatment of AD, because the compound can act as an endogenous anti-oxidant, free radical- and metal ion chelator, and also has neuroprotective activity against in vitro Aβ-induced toxicity [20], [32], [37]. Thus, the major aim of this study was to evaluate the effect of dietary carnosine supplementation in a model of AD that develops an age-related neurodegenerative phenotype that is driven by intraneuronal deposition of Aβ and accumulation of h-tau [11]. We chose to treat only male 3xTg-AD mice as female hormones are known to negatively influence the activity of Zn^2+^ transporters [38] and differently affect the disease progression [39], therefore producing confounding effects. We chose to use PS1-KI animals as control group as these mice overexpress mutant Presenilin-1 gene (M146V substitution) but, by lacking the expression of mutant APP and h-tau, do not show Aβ or tau-dependent pathology nor AD-related cognitive deficits [30].

Carnosine has been shown to protect against Zn^2+^-mediated toxicity in cell cultures and that activity has been linked to the chelating properties of the compound. The peptide has indeed been shown to complex Zn^2+^ in acqueous solution but, to date, there were no experimental data demonstrating its chelating capability in biological systems. We tested this hypothesis in cultured glial cells and show that the peptide is in fact able to chelate [Zn^2+^]_i_ (Figure 1).

In our study, we also found a very potent effect of carnosine in rescuing mitochondrial dysfunctions in aged 3xTg-AD mice. As discussed above, mitochondrial deficits are emerging as key players in AD [3] [40] and, in line with observations indicating that Aβ and tau synergistically impair OXPHOS complexes [4], we found signs of potent deregulation of mitochondrial respiration in our AD mice. In 3xTg-AD mice at 12–14 m.o.a., we observed a reduction in the activity of complex I, II and IV in the hippocampus as well as of complex I and IV in the cortex. Interestingly, carnosine supplementation not only prevented such deficits but, in the hippocampus, we found that complexes II and IV activity of carnosine-fed AD mice actually increased over baseline (Figure 4). Such results can be linked to the antioxidant activity of the dipeptide, a property that can prevent ROS-dependent mobilization of [Zn^2+^]_i_. Such activity can block Zn^2+^-dependent mitochondrial dysfunction [34], and inhibit the overproduction of nitric oxide [41], a process strongly potentiated by Zn^2+^ that eventually contributes to a self perpetuating mobilization of the cation. In addition, carnosine may have a direct effect on Aβ deposition and mitochondrial function by acting as an osmolyte as shown in the case of its action on methylene blue and cytochrome c oxidase [42].

When we analyzed the effects of carnosine on amyloid and tau pathology we found that the peptide is very effective in decreasing intraneuronal Aβ deposition in the hippocampus but does not affect the development of tau pathology.

Analysis of the effect of carnosine supplementation on cognitive deficits of 3xTg-AD mice showed a positive trend, indicating that it might have a beneficial role in preventing long-term memory deficits, although, this effect did not reach statistical significance. The sub-maximal effect we observed could be related to the fact that carnosine is able to greatly inhibit the Aβ load but not the appearance of tau pathology and these two molecular components are definitely acting synergistically in the development of the cognitive decline [43], [44], [45].

In the last few years a growing body of evidence is supporting the intriguing hypothesis that the alteration in the equilibrium of brain Zn^2+^ levels can be a significant contributing factor for AD [6]. Interestingly, both excess as well as deficit of brain Zn^2+^ can favour AD-like pathology in AD animal models [36], [46] suggesting the existence of a finely tuned Zn^2+^ set point. Such hypothesis has been substantiated by a recent study indicating that deficits of synaptic Zn^2+^ promotes AD-like cognitive impairment by negatively interfering with glutamatergic and BDNF signalling [47]. Thus, in such scenario, a decreased Zn^2+^ bioavailability induced by carnosine supplementation may in part exert negative effects on the neurotransmission and neurotrophic signalling that modulate cognitive functions, and in doing so, counteracts the positive activity on amyloid deposition and mitochondrial functioning. In that respect, it could be interesting to verify the possibility that more effective synergistic activity can be achieved when carnosine and Zn^2+^ are administered together in a combined form similarly to what has been described for the compound named Polaprezinc, a preparation that combines Zn^2+^ and L-carnosine [48], [49]. In theory, this compound, given the different K_d_ for Zn^2+^ of carnosine and Aβ can act as an homeostatic molecule that sequesters Zn^2+^ from Aβ but then releases a sufficient amount of the cation in the synaptic cleft to exert neurotrophic actions.

Another possibility that could explain the subthreshold effect on cognition is associated with changes in carnosinase activity as the enzyme has been found to undergo an age-dependent enhanced activity in brains of aging individuals and AD patients [28], [29]. Finally, it is also possible that a more robust effect could be revealed by extending these behavioural studies to a larger cohort of animals.

In summary, carnosine has a strong effect in restoring mitochondrial functioning and in counteracting amyloid pathology but these activities do not translate in a robust effect on cognition. These results suggest that, at least in complex AD animal models, addressing mitochondrial dysfunction and Aβ aggregation without a parallel intervention on h-tau deposition is not sufficient to promote major beneficial cognitive effects. Supporting this idea, recent reports have in fact indicated that therapeutic measures addressing Aβ overloads but unable to reduce the development of tau pathology do not prevent the development of cognitive deficits in 3xTg AD mice [44], [50].

## Materials and Methods

### Materials

Newport Green and pluronic acid were purchased from Molecular Probe (Invitrogen). L-Carnosine and DTDP (2,2′-dithiodipyridine) were obtained from Sigma Aldrich. Tissue culture media and serum were purchased from Gibco (Invitrogen).

### Glial cell cultures

Murine cortical glial cultures were prepared from CD-1 mice as previously described [51]. Briefly, neocortices from 1–3 day pups were dissociated and plated in 35 mm glass bottom dishes in a MEM medium in the presence of 10% horse serum and 10% fetal bovine serum. Cells were maintained in a humidified atmosphere containing 5% CO_2_ and used for the experiments 10 days after plating.

### Zn^2+^ imaging

Zn^2+^ imaging was performed using an inverted microscope (Nikon Eclipse TE 300) equipped with a xenon lamp, a filter wheel (Lambda shutter 10–2, Sutter Instruments), and a 40X epifluorescence oil immersion objective. Glial cells were loaded in the dark with the DCF diacetate form of the Zn^2+^-sensitive probe Newport Green (5 µM + 0.2% of Pluronic Acid), in a HEPES-buffered medium (HCSS) whose composition was (in mM): 120 NaCl, 5.4 KCl, 0.8 MgCl_2_, 20 HEPES, 15 glucose, 1.8 CaCl_2_, 10 NaOH, pH 7.4 for 30 min at 25°C. Cultures were then washed in HCSS and kept in the dark for an additional 30 min. Excitation was at 490 nm, with emission at 510 nm as previously described [8]. Images were acquired every 30 sec during all the experimental session. To compensate for cell-to-cell variability in dye loading, after background subtraction from a cell-free region of the dish, Newport Green fluorescence measurements for each cell (F_x_) were normalized to the fluorescence intensity for that cell at the beginning of the experiment (F_0_). Drugs were applied by bath application and removed through a rapid flow exchange system. Images were acquired with a 12-bit digital CCD camera (Orca, Hamamatsu) and analyzed with Metafluor 6.0 software (Invitrogen). Values are reported as mean ± SEM of FluoZin-3 F_x_/F_0_ ratios.

### Animals and treatment paradigm

Procedures involving animals and their care were approved by the institutional Ethics Committee (CeSI protocol #: AD-301) and conducted in conformity with the institutional guidelines that are in compliance with national (D.L. n. 116, G.U., suppl. 40, 18 February 1992) and international laws and policies. All efforts were made to minimize the number of animals used and their suffering. Transgenic mice were characterized and described by Oddo et al. [11] and generously provided by Frank Laferla. One month old male 3xTg-AD mice (n = 9) were treated with 10 mM L-Carnosine (Sigma-Aldrich) in standard tap water for a period of 11–13 months. Control groups [3xTg-AD (n = 13) and control PS1-KI (n = 11)] mice were given just tap water.

### Morris water maze

The Morris water maze (MWM) apparatus consisted of a circular plastic tank filled with water (1.3 m diameter). The maze was located in a room containing several intra and extra-maze visual cues. Mice were trained to swim on a (12×13 cm) rectangular platform submerged 2 cm beneath the surface of the water and invisible to the animals while swimming. To reduce stress, mice were placed on the platform 10 s prior to the first training trial. They were allowed to find an escape by climbing on the submerged platform; if the mouse failed in finding the platform within 90 s, it was manually guided to the platform and allowed to remain there for 10 s. After that period, each mouse was placed into a holding cage under a warming fan for 20 minutes, until the start of the next training trial. Mice were given 4 trials per day for 3 consecutive days with an inter-trial time of 20 min. Retention of the spatial memory was assessed 1.5 and 24 hours after the end of the last training trial. Both probe trials consisted of a 60 s free swim in the pool without the platform. Mice were monitored by a digital camera mounted on the ceiling of the room directly above the pool and all trials stored for subsequent analysis. The parameter employed to evaluate memory skills was the time (latency) to reach and cross the platform location.

### Immunohistochemistry

Carnoy-fixed and paraffin embedded brains were sagittaly sectioned (n = 3 per group). Antigen retrieval was performed by microwave treatment at 750 W for 10 min in a 10 mM sodium citrate buffer (pH 6.0). After blocking the endogenous mouse IgG antibodies (Biocare Medical), sections were incubated overnight with the primary antibody. Slices were then incubated with the secondary anti-mouse (HRP-polymer, EnVision kit, Dako), counterstained with Mayer's hematoxylin, and the reaction visualized using diamminobenzidine as chromogen. The number of stained pyramidal neurons and neurofibrillary tangles was measured using Photoshop 8.0 (Adobe Systems Incorporated) by using the Photoshop Lasso Tool and pixel numbers obtained from the resulting histogram. After this first step, we used the Magic Wand tool to select a representative positive cell signal. All the immunostained cells were automatically selected and the total pixel number recorded. Pixel counts were normalized to a hippocampal area of 1 mm^2^.

### Antibodies

The following antibodies were used: anti-Aβ, clone DE2B4, diluted 1∶400 (Abcam); anti-phosphoTau,clone AT180, 1∶400 (Pierce).

### Mitochondrial sample preparation

Samples for Blue Native-Poly-Acrylamide Gel Electrophoresis (BN-PAGE) were prepared as described in detail by Schägger with minor modifications [52]. AD and control mice were killed by decapitation. Brains were quickly dissected on ice; cortex and hyppocampus were immediately frozen on dry ice and stored at −80°C until use. Brain tissues (10 mg; wet weight) were homogenized in BN-sample buffer 1 (250 mM Sucrose, 30 mM morpholine-propane sulfonate buffer, 0.2 mM phenylmethylsulfonyl fluoride, pH 7.2) using a homogenizer with a tight-fitting Teflon pestle (1 min at 500 rpm) and kept cold by immersing the homogenizer in an ice-filled beaker. The homogenates were centrifuged at 20,000 g for 20 min at 4°C and the supernatants were discarded. To solubilize mitochondrial membranes, mitochondria-rich pellets were vigorously pipetted in BN-sample buffer 2 (1 M aminocaproic acid, 50 mM Bis-Tris-HCl buffer, pH 7) and homogenized by twirling with a tiny spatula. Next, freshly prepared 10% dodecyl maltoside was added to mitochondria containing sediment to solubilize individual respiratory chain complexes. The homogenates were centrifuged at 20,000 g for 1 h at 4°C. The supernatants were collected and transferred intto a new tube. The gel loading mixture was prepared by adding 5% Coomassie Blue Brilliant G-250 dissolved in 1 M aminocaproic acid to the sample at a ratio of 1∶6 (dye:sample volume).

### BN-PAGE

After solubilization of mitochondrial membranes by dodecyl maltoside, BN-PAGE, staining and densitometric quantification of oxidative phosphorylation complexes were performed essentially as described by Zerbetto [53]. Briefly, samples were applied to a 6–13% gradient acrylamide gel with a 4% polyacrylamide stacking gel. To assure the same conditions, gels were made simultaneously using Mini-Protein II Multi-gel casting chamber (Bio-Rad). Twenty micrograms of each sample were loaded at 4°C into 5×1.5 mm gel wells and were at first run until they have entered the stacking gel, typically 80 V for 30–40 min, after which the voltage was increased to 100 V and the blue cathode buffer was replaced by an uncoloured buffer. Electrophoresis continued until the blue dye front reached the end of the gel. Immediately after electrophoresis, the gels were fixed in 40% methanol and stained with a Coomassie Blue Solution (Brillant Blue G and methanol used at a ratio of 1∶4 respectively) overnight for measuring the amount of proteins. The next day, the gels were destained with 10% acetic acid and 25% methanol for 1–2 min and then washed different times with 25% methanol. For the histochemical evaluation of BN-PAGE of each set of gels, one gel was stained with Coomassie Blue (as described above), other gels were used for determining specific enzyme activity by the following reactions. Complex I (NADH-Dehydrogenase) activity was determined by incubating the gel with 0.1 M Tris–HCl, 768 mM glycine, 0.1 mM β-NADH and 0.04% Nitro Blue Tetrazolium (NTB) pH 7.4 at room temperature (RT). As a measure for complex II activity, Succinate Dehydrogenase (SDH) activity was determined as follows. The gel was incubated in 0.1 M Tris–HCl, 100 mM glycine, 10 mM succinic acid and 1 mg/ml NTB pH 7.4 at RT. Complex IV (COX) activity was estimated by incubating the gels with 5 mg of 3,3′–diaminobenzidine tetrahydrochloride (DAB) dissolved in 9 ml phosphate buffer (0.05 M pH 7.4), 1 ml catalase (20 µg/ml), 10 mg cytocrome C and 750 mg sucrose. Violet colored complex I and complex II bands and red-stained complex IV were measured using the Bio-Rad Imaging Densitometer (Quantity One Analysis Software, BioRad) with a blue filter inserted to minimize interference from the residual Coomassie Blue. The band intensities were expressed as absolute values (arbitrary units; AU). Optical densities (OD) of the bands from each loading amount were plotted against the respective value determined in Coomassie Blue gel.

### Statistic analysis

Statistical differences were determined with the Student's t-test for unpaired data. For analysis of the MWM latencies and BN-PAGE experiments, the homogeneity of the variances was determined by the Bartlett test (90% confidence level) and a one-way ANOVA was performed followed by a post hoc Bonferroni's correction. For immunohistochemistry experiments, Mann-Whitney test was employed. All data are expressed as mean ± SEM and the threshold for statistically significant differences was set at P<0.05.
